# MZF1 Transcriptionally Activated MicroRNA-328-3p Suppresses the Malignancy of Stomach Adenocarcinoma via Inhibiting CD44

**DOI:** 10.1155/2022/5819295

**Published:** 2022-05-28

**Authors:** Zining Qi, Jing Wang, Yaoping Li, Yanzhao Xu

**Affiliations:** ^1^Department of Gastrointestinal Surgery, The First Hospital of Shanxi Medical University, Taiyuan, Shanxi, China; ^2^Department of Head and Neck Surgery, Shanxi Cancer Hospital, Taiyuan, Shanxi, China; ^3^Department of Colorectal Anorectal Surgery, Shanxi Provincial People's Hospital, Taiyuan, Shanxi, China; ^4^Department of Thoracic Surgery, The Fourth Hospital of Hebei Medical University, Shijiazhuang, Hebei, China

## Abstract

MicroRNA-328-3p (miR-328-3p) plays a critical role in mediating the progression of multiple types of cancers. To date, no study has concentrated on the molecular mechanism of miR-328-3p in mediating stomach adenocarcinoma (STAD). In this study, it was found that miR-328-3p was downregulated in STAD, and inhibition of miR-328-3p significantly promoted the growth, migration, invasion, and stemness of STAD cells, while miR-328-3p overexpression exerted reverse effects. Through bioinformatics analysis, it was uncovered that a cluster of differentiation 44 (CD44) was upregulated in STAD and closely associated with the prognosis of STAD patients. Mechanistically, we identified CD44 as the target gene of miR-328-3p. Notably, knockdown of CD44 abolished the promoting function of miR-328-3p inhibitor in the development of STAD. Moreover, myeloid zinc finger protein 1 (MZF1) was confirmed as an upstream transcription factor for miR-328-3p, which is involved in enhancing miR-328-3p expression. In addition, the role of MZF1 downregulation in the malignant traits of STAD cells was blocked by miR-328-3p overexpression. More importantly, upregulation of miR-328-3p efficiently suppressed STAD tumor growth *in vivo*. Collectively, our findings illustrated that MZF1-mediated miR-328-3p acted as a cancer suppressor in STAD progression via regulation of CD44, which suggested the possibility of the MZF1/miR-328-3p/CD44 axis as a novel promising therapeutic candidate for STAD.

## 1. Introduction

Gastric cancer (GC) is a prevalent and invasive malignancy in the digestive system with poor prognosis [1]. Stomach adenocarcinoma (STAD) is identified as the most common histopathological type of GC [2], accounting for 90% of all GC cases worldwide [3]. In addition, STAD is characterized by rapid proliferation and metastasis [2], which is associated with poor prognosis of STAD patients. Cancer stem cells can self-renew and differentiate and play a critical role in the tumorigenesis and therapeutic resistance of GC, including STAD [4, 5]. The stemness of cancer cells is of great significance in tumor development, metastasis, and recurrence [6]. The superficial understanding of the molecular mechanism underlying STAD tumorigenesis has restricted the development of effective therapeutic strategies of STAD, and novel biomarkers for predicting STAD recurrence are urgently required to ameliorate its prognosis.

MicroRNAs (miRNAs) are small noncoding single-stranded RNA molecules with approximately 19–23 nucleotides [7], which exert repressive or degradable effects on messenger RNA via binding to its 3′-untranslated region [8]. As a member of miRNAs, microRNA-328-3p (miR-328-3p) has been proved to regulate the progression of diverse malignant tumors. For instance, Liu et al. suggested that hypoxic bone marrow mesenchymal cell-extracellular vesicles containing miR-328-3p promote lung cancer progression via the NF2-mediated Hippo axis [9]. Pan et al. reported the inhibitory effects of miR-328-3p on growth and metastasis of colorectal cancer cells via binding to Girdin [10]. In addition, Yi et al. revealed the antiproliferative effects of bioengineered miR-328-3p on human osteosarcoma cells via regulating GLUT1-mediated glucose uptake [11]. Intriguingly, a recent study reported that miR-328-3p was remarkably downregulated in STAD tissues, and it could suppress the cell proliferation through binding to signal transducer and activator of transcription 3 (STAT3) [12]. However, other fundamental mechanisms and functions of miR-328-3p in STAD are still elusive.

Cluster of differentiation 44 (CD44) is a glycoprotein [13], and it is regarded as a receptor for hyaluronic acid, owning to its capacity of binding to hyaluronic acid by its amino-terminal domain [14]. Based on previously reported data, CD44 has been proven to engage in the interaction between cells and the extracellular matrix (ECM) [15, 16] and participate in the lymphocyte homing and the activation of T cells [17]. Moreover, CD44 is highly associated with tumorigenicity, invasiveness, and lymphatic metastasis of tumor cells [18]. Specifically, Roshani et al. demonstrated that downregulating CD44 suppressed the capacities of colony and spheroid formation of colorectal cancer cells [19]. Zhang et al. pointed out that the nuclear factor-*κ*B (NF-*κ*B)/CD44 pathway accelerated the tumor growth and metastasis of bladder carcinoma [20]. In addition, as a biomarker of gastric cancer stem cells (GCSCs), CD44 is involved in tumor growth and maintenance of cancer cell stemness [21]. For instance, Sohn et al. reported that foretinib represented a promising agent for the prevention or treatment of GCs positive for CD44 variant 9 (CD44v9) and c-mesenchymal-epithelial transition (c-MET) [22]. Nevertheless, the mechanism by which miR-328-3p mediates CD44 to regulate STAD remains unclear.

Myeloid zinc finger protein 1 (MZF1) is encoded by the MZF1 gene on human chromosome 19 [23] and is a member of the SCAN domain-containing zinc finger transcription factor (SCAN-ZFP) family [24]. As a transcription factor, MZF1 is characterized by a unique zinc finger structure and can bind to the promoter regions of target genes [25]. According to previous studies, MZF1 participates in the progression of diverse types of cancers via transcriptionally activating target genes [26, 27]. Additionally, MZF1 depletion repressed the transcription of CDC37 and impeded the tumorigenesis of prostate cancer [25], and MZF1 induced the transcriptional activities of c-Myc to mediate the progression of gliomas [28]. Recently, Lin et al. suggested that metallothionein 2A (MT2A) exerted its antigastric cancer effects via MZF1 [29]. However, the role of MZF1 in transcriptional regulation of miRNA in STAD remains elusive.

Therefore, we detected the expression level of miR-328-3p in STAD and investigated its effects on the behaviors of STAD cells in the present research. Moreover, using data collected from online databases and cellular experiments, we explored the correlation between miR-328-3p and CD44, as well as MZF1 and miR-328-3p, and determined the functional role of the MZF1/miR-328-3p/CD44 axis in regulating STAD.

## 2. Materials and Methods

### 2.1. Cell Culture and Transfection

Human STAD cell lines)HGC-27, AGS, and SNU16) and normal gastric mucosa cell line GES-1 were purchased from the American Type Culture Collection (ATCC; Manassas, VA, USA) and cultured in a Roswell Park Memorial Institute- (RPMI-) 1640 medium (catalog no. 11875119; Gibco, Rockville, MD, USA) containing 10% fetal bovine serum (FBS; catalog no. S-FBS-IR-015; Serana, Berlin, Germany) at 37°C under 5% CO_2_. The pcDNA-CD44, miR-328-3p-mimics, siRNAs targeting CD44 (si-CD44), siRNAs targeting MZF1 (si-MZF1), and miR-328-3p inhibitors were provided by GeneChem Co., Ltd. (Shanghai, China) (Supplementary Table 1). STAD cells were transfected with indicated plasmids using Lipofectamine 2000 (catalog no. 11668-019; Invitrogen, Carlsbad, CA, USA) and were cultured for 48 h for further research.

### 2.2. Cell Counting Kit-8 (CCK-8) Assay

To evaluate the cell proliferation rate, CCK-8 assay was employed. In brief, AGS and SNU16 cells were added into 96-well plates for incubation. After 24-, 48-, and 72-hour cultivation at 37°C, cells were treated with 10% CCK-8 solution (catalog no. 96992; Sigma-Aldrich, St. Louis, MO, USA) for additional 2 h at 37°C, respectively, and the absorbance at 450 nm was measured using a microplate reader (Multiskan SkyHigh; Thermo Fisher Scientific, Waltham, MA, USA).

### 2.3. Ethylenediurea (EdU) Assay

After being seeded into 24-well plates, the pretreated AGS and SNU16 cells were stained using an EdU staining kit (catalog no. C10337; Thermo Fisher Scientific) according to the manufacturer's instructions. Following fixation with 4% paraformaldehyde for 15 min, images were captured using an inverted fluorescence microscope (DMI6000 B, Leica Microsystems, Heidelberg, Germany), and the number of EdU-positive cells was counted under randomly selected fields.

### 2.4. Transwell Invasion Assay

To assess the invasion of AGS and SNU16 cells, Transwell assay was conducted. 3 × 10^4^ cells in 200 *μ*l serum-free medium were added to the upper chamber precoated with Matrigel gel (catalog no. 354234; BD Biosciences, San Jose, CA, USA). Then, 500 *μ*l of 10% FBS-containing medium was added to the lower chamber as a chemoattractant. Cells were incubated for 48 h at 37°C, and then, cells on the surface of the filter membrane were removed with cotton swabs. Afterwards, the invaded cells were fixed with 4% paraformaldehyde (catalog no. P6148; Sigma-Aldrich) for 30 min, stained with 0.04% crystal violet (catalog no. C0775; Sigma-Aldrich) for 10 min, and counted using an optical microscope (Primotech; Zeiss, Berlin, Germany).

### 2.5. Wound Healing Assay

Wound healing assay was performed to assess and quantify collective cell migration under different experimental conditions. In brief, a sterile pipette tip was used to finish scratch operation on AGS and SNU16 cells. Then, the images were captured using a microscope, and the Image-Pro Plus 6.0 software was adopted to measure the wound area.

### 2.6. Dual-Luciferase Reporter Assay

Data collected from TargetScan and TransmiR databases were, respectively, analyzed for prediction of the binding sites between miR-328-3p and CD44 and between MZF1 and miR-328-3p. For dual-luciferase reporter analysis, cotransfection of miR-328-3p mimics or negative control (NC) mimics was performed with CD44-MUT and CD44-WT. Meanwhile, MZF1-overexpressing vectors and empty vectors were cotransfected into AGS and SNU16 cells with miR-328-3p-MUT and miR-328-3p-WT, respectively. The Dual-luciferase Reporter Assay System (catalog no. E1910; Promega Inc., Madison, WI, USA) was employed for the detection of luciferase activity.

### 2.7. Chromatin Immunoprecipitation (ChIP) Assay

ChIP assay was conducted using the Chromatin Immunoprecipitation Kit (catalog no. 17–10,086, Merck Millipore, Burlington, MA, USA). AGS and SNU16 cells were first fixed with 1% formaldehyde (catalog no. 252549; Sigma-Aldrich) for 10 min, followed by sonication for the acquisition of DNA fragments. Protein A/G agarose (catalog no. IP10, Merck Millipore) was used to eliminate the lysate and then incubated with anti-MZF1 (catalog no. ab64866; Abcam, Cambridge, UK) or IgG (catalog no. ab171870; Abcam) antibodies at 4°C overnight. A QIA Quick Purification Kit (catalog no. 28104; Qiagen, Hilden, Germany) was utilized to release and purify the DNA fragments, and then, the quantitative reverse transcription polymerase chain reaction (qRT-PCR) was performed on the immunoprecipitated DNA fragments.

### 2.8. Tumor Xenograft Experiment

For the construction of a xenograft mouse model, a total of 12 BALB/c athymic male nude mice (age, 5-week-old; weight, 20 g) were obtained from Beijing Huafukang Bioscience Co., Ltd. (Beijing, China) and were raised in specific-pathogen-free (SPF) conditions with 40-60% humidity at 28°C under 12 h/12 h light/dark cycle. SNU16 cells were subcutaneously injected into the flanks of nude mice, and subsequently, the intratumor injection of miR-328-3p-agomir or agomir-NC was performed. After 28 days of feeding, nude mice were executed mercifully, and their xenografts were dissected and weighed. Tumor size was assessed by measurement of the length (*L*) and width (*W*) of the tumors in two dimensions using a Vernier caliper as soon as tumors reached a measurable size. All animal experiments were approved by the Animal Care and Use Committee of Fourth Hospital of Hebei Medical University (Shijiazhuang, China).

### 2.9. Immunohistochemistry (IHC)

The xenograft tumor samples were fixed with 4% paraformaldehyde, dehydrated with ethanol, and were embedded into paraffin wax. IHC was used to detect Ki67 and CD44. Briefly, Ki67 (catalog no. ab15580; Abcam) and CD44 (catalog no. ab189524; dilution, 1 : 4,000; Abcam) primary antibodies were added and were mixed with the secondary antibodies labeled with biotin for 10 min. Then, the 3,3′-diaminobenzidine substrate (DAB) staining was carried out. Optical microscopy was performed for further analysis.

### 2.10. Terminal Deoxynucleotidyl Transferase dUTP Nick End Labeling (TUNEL) Staining

TUNEL assay was performed for the estimation of cell apoptosis in tumor tissues obeying the directions of an In Situ Cell Death Detection Kit (catalog no. 11684817910; Roche, Germany). In short, tissue sections were deparaffinized, rehydrated, permeabilized, and stained by TUNEL reaction reagent at 37°C for 1 h in the dark. After treatment with converter POD for 30 min at 37°C, sections underwent DAB staining and then were counterstained with hematoxylin. Images were obtained using optical microscopy.

### 2.11. Western Blot Assay

For the extraction of total protein from STAD cells, radioimmunoprecipitation assay (RIPA) lysis buffer (catalog no. P0013B; Beyotime, Shanghai, China) was utilized, and polyacrylamide gel electrophoresis (PAGE) gel (catalog no. M01215C; Jinsirui, Nanjing, China) was used to isolate the protein samples, which were then transferred onto polyvinylidene fluoride (PVDF) membranes (catalog no. 03010040001; Roche,), followed by incubation with primary antibodies at 4°C overnight. The secondary antibody goat anti-rabbit IgG conjugated to horseradish peroxidase (HRP) (catalog no. ab205719 or ab205718; Cell Signaling Technology, Dallas, TX, USA) was mixed with the membranes, and the ImageJ software (National Institutes of Health, Bethesda, MD, USA) was employed for quantification of protein expression. Rabbit anti-glyceraldehyde 3-phosphate dehydrogenase (GAPDH) antibody (catalog no. ab9485; dilution, 1 : 1000; Abcam) was used as an internal control. Besides, rabbit anti-Bax antibody (catalog no. ab32503; dilution, 1 : 2,000), rabbit anti-Bcl-2 antibody (catalog no. ab32124; dilution, 1 : 1,000), rabbit anti-caspase-3 antibody (catalog no. ab32351; dilution, 1 : 5,000), rabbit anti-cleaved caspase-3 antibody (catalog no. ab32042; dilution, 1 : 500), rabbit anti-caspase-9 antibody (catalog no. ab32539; dilution, 1 : 1,000), rabbit anti-cleaved caspase-9 antibody (catalog no. ab2324, dilution, 1 *μ*g/ml), and rabbit anti-MZF1 antibody (catalog no. ab64866, dilution, 1 : 1,000) were all purchased from Abcam.

### 2.12. qRT-PCR

qRT-PCR was performed to determine the expression levels of miR-328-3p, CD44, and MZF1. In brief, TRIzol reagent (catalog no. T9424; Sigma-Aldrich) was used for the extraction of total RNA from STAD cells. Then, qRT-PCR analysis was carried out using the ABI 7300 real-time PCR system (ABI 7300; Applied Biosystems, Waltham, MA, USA). The 2^−*ΔΔ*Ct^ method was utilized to determine the relative expression. In this experiment, the primer sequences were designed by Sangon Biotech Co., Ltd. (Shanghai, China), as presented in Supplementary Table 2.

### 2.13. Bioinformatics Analysis

To identify the expression pattern of miR-328-3p in GC tissues, we analyzed raw data downloaded from NCBI GEO (GSE33743) and The Cancer Genome Atlas (TCGA) databases. The expression data of miR-328-3p was acquired from the downloaded raw data and then, Student's *t*-test was carried out to determine the difference of miR-328-3p expression between two groups. Through *P* < 0.05 and log fold change (FC) > 1.0, upregulated mRNAs with differential expression in STAD were screened out by using the GEPIA2 database. The potential target genes of miR-328-3p were predicted by miRTarBase. The overlapped mRNAs from GEPIA2 and miRTarBase were identified using Venn diagram. Similarly, the common transcription factors of miR-328-3p in PuTmiR and TransmiR databases were found by Venn diagram. In addition, univariate and multivariate Cox regression analyses were performed to estimate the prognostic role of gene expression and clinical parameters extracted from the TCGA dataset. The Kaplan-Meier plotter was applied to evaluate the prognostic role of CD44 for the survival of STAD patients based on the best cutoff. Besides, the expression pattern of four common transcription factors (MZF1, RREB1, BACH1, and STAT1) in STAD and noncancer tissues was identified by the GEPIA2 database. Meanwhile, Oncomine database was employed to reveal the expression of MZF1 and CD44 in GC. GO enrichment analysis was performed to explore the coexpressed genes of CD44 using “clusterProfiler” package in R 3.14.3 programming language, and the data were normalized using a cutoff value adjusted to *P* < 0.05.

### 2.14. Identification of Bottleneck Genes and Hub Genes

A protein-protein interaction (PPI) network was established by uploading the overlapped genes to the STRING online database, and the top 10 hub genes were selected through the algorithm of degree in cytoHubba plugin of Cytoscape. Through another important algorithm bottleneck, the top 10 bottleneck nodes in this network were identified.

### 2.15. Statistical Analysis

The SPSS 20.0 software (IBM, Armonk, NY, USA) was utilized to perform the statistical analysis, and figures were drawn using the GraphPad Prism 7.0 software (GraphPad Software Inc., San Diego, CA, USA). Student's *t*-test and one-way analysis of variance (ANOVA) with post hoc test were employed to compare differences between two or multiple groups for data obeying a normal distribution. The overall survival was assessed using the Kaplan-Meier method. Data were expressed as the mean ± standard deviation (SD). *P* < 0.05 was considered statistically significant.

## 3. Results

### 3.1. miR-328-3p Was Downregulated in STAD

**Figure 1:**
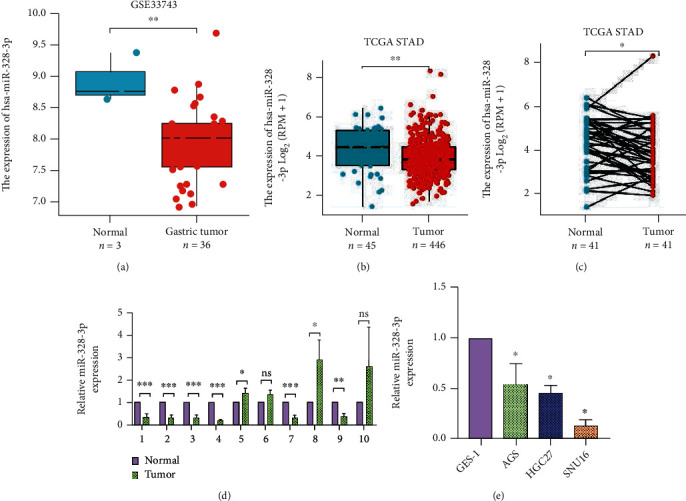
Low expression of miR-328-3p in STAD cells and tissues. (a) The expression pattern of miR-328-3p in GC via analyzing GSE33743 downloaded from NCBI GEO. (b, c) TCGA database was used to evaluate the expression level of miR-328-3p in (b) unpaired and (c) matched STAD samples. (d) qRT-PCR analysis of miR-328-3p expression in 10 pairs of clinical samples. (e) qRT-PCR was used to detect the expression level of miR-328-3p in STAD cells (AGS, HGC27, and SNU16) and in normal cells GES-1. All experiments were performed in triplicate. ^∗^*P* < 0.05, ^∗∗^*P* < 0.01, and ^∗∗∗^*P* < 0.001.

First, the expression pattern of miR-328-3p in gastric cancer was identified through employment of GSE33743 downloaded from NCBI GEO. Data disclosed that the miR-328-3p level in GC samples was lower than that in normal specimens (Figure 1(a)). Next, we explored the miR-328-3p expression level in unpaired (Figure 1(b)) and matched (Figure 1(c)) STAD tissue samples collected from The Cancer Genome Atlas (TCGA) database. As expected, it was found that miR-328-3p was significantly downregulated in STAD tissues compared with normal tissues. Consistently, the qRT-PCR assay further confirmed downregulation of miR-328-3p in clinical STAD tissues (Figure 1(d)). Likewise, we observed that the miR-328-3p expression level was significantly lower in STAD cells than in normal cells GES-1 (Figure 1(e)). Collectively, miR-328-3p expression levels were downregulated in STAD.

### 3.2. miR-328-3p Suppressed the Malignant Behaviors of STAD Cells

**Figure 2:**
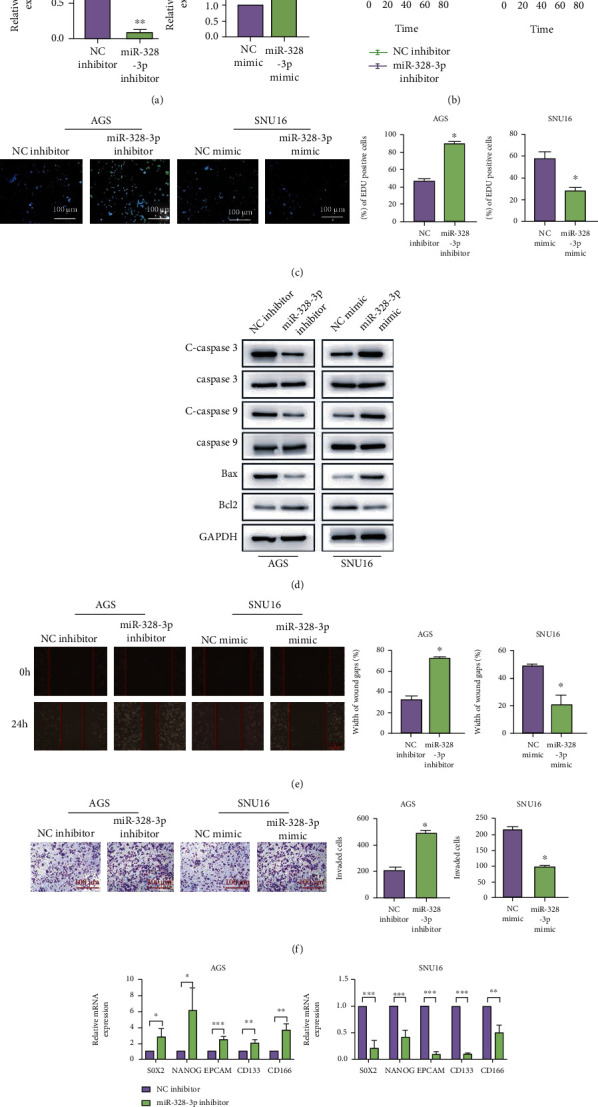
miR-328-3p suppressed the malignant features of STAD cells. To evaluate the functional role of miR-328-3p in STAD, AGS cells were transfected with miR-328-3p inhibitors and SNU16 cells were treated with miR-328-3p mimics. (a) The transfection efficiency of miR-328-3p detected by qRT-PCR. (b, c) The proliferation of AGS and SNU16 cells was detected with (b) CCK-8 and (c) EdU assays. (d) Western blot assay was employed to detect the expression levels of apoptosis-related proteins. (e, f) The migration and invasion of STAD cells were assessed by (e) wound healing and (f) Transwell assays. (g) qRT-PCR analysis of the expression levels of stem landmarks. Scale bar = 100 *μ*m. All experiments were performed in triplicate. ^∗^*P* < 0.05, ^∗∗^*P* < 0.01, and ^∗∗∗^*P* < 0.001.

To further explore the biological effects of miR-328-3p on STAD, we knocked down miR-328-3p in AGS cells and overexpressed miR-328-3p in SNU16 cells by virtue of miR-328-3p mimics and inhibitors (Figure 2(a)). CCK-8 assay showed that silencing miR-328-3p elevated the viability of AGS cells, whereas enhanced expression of miR-328-3p repressed the proliferation of SNU16 cells (Figure 2(b)). Similarly, results of EdU staining validated the inhibitory function of miR-328-3p in STAD cell proliferation (Figure 2(c)). In addition, Western blot illustrated that knockdown of miR-328-3p increased the expression of antiapoptotic protein Bcl-2 and reduced the protein levels of proapoptotic factors Bax, cleaved caspase-3, and cleaved caspase-9. Meanwhile, upregulation of miR-328-3p triggered opposite effects (Figure 2(d)). In concert with these results, wound healing and Transwell assays revealed that inhibition of miR-328-3p enhanced the migratory and invasive capacities of STAD cells, while miR-328-3p overexpression suppressed SNU16 cell migration and invasion (Figures 2(e) and 2(f)). Moreover, it was observed that downregulation of miR-328-3p elicited a prominent increase in the mRNA expression of stem cell markers SOX2, NANOG, EPCAM, CD133, and CD166 and overexpression of miR-328-3p reduced levels of these landmarks (Figure 2(g)). Taken together, miR-328-3p acted as a cancer suppressor in STAD.

### 3.3. CD44 Expression Level Was Negatively Regulated by miR-328-3p in STAD Cells

**Figure 3:**
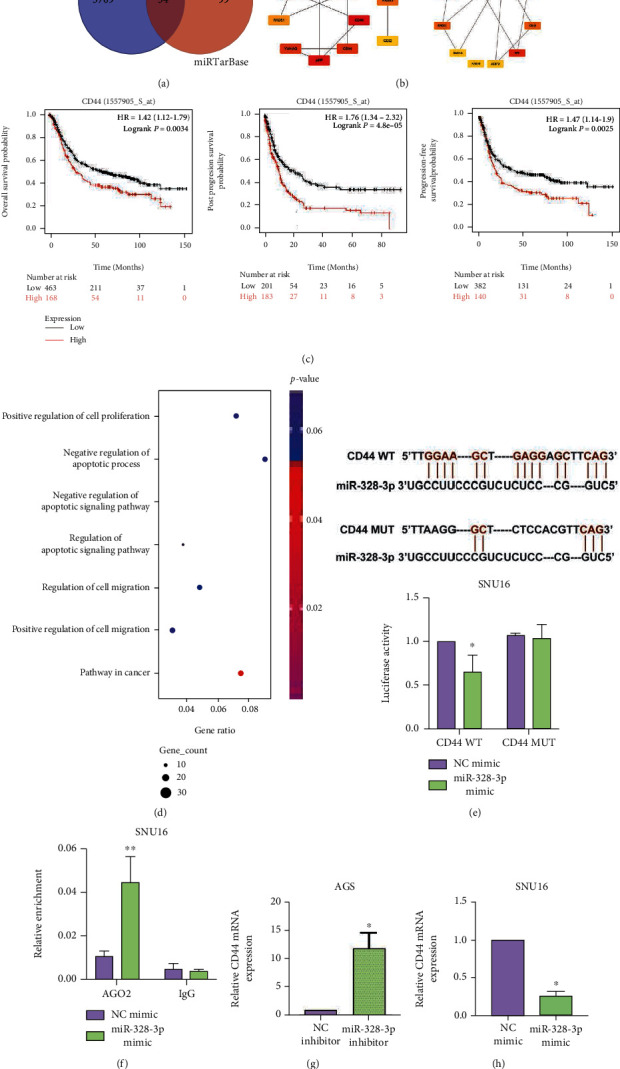
miR-328-3p negatively modulated CD44 expression. (a) The GEPIA2 and miRTarBase databases were used to identify overlapped genes. (b) The top 10 bottleneck proteins and top 10 hub proteins were selected by utilization of bottleneck and degree algorithm, respectively. (c) The association between CD44 and the prognosis of STAD patients. (d) The CD44 coexpressed genes were predicted to be enriched in 7 GO terms. The *x*-axis represented the gene ratio, and the intensities of different colors represented the adjusted *P* value. (e) The binding sites between miR-328-3p and CD44 predicted by TarBase database were shown. Dual-luciferase reporter assay was employed to evaluate their interaction. (f) RIP assay was also performed to verify the relationship between miR-328-3p and CD44. (g, h). qRT-PCR was utilized to detect the expression level of CD44 in (g) miR-328-3p-downregulated AGS cells and (h) miR-328-3p-overexpressed SNU16 cells. All experiments were performed in triplicate. ^∗^*P* < 0.05, ^∗∗^*P* < 0.01.

To investigate the mechanism of miR-328-3p in regulating the biological function of STAD cells, bioinformatics analysis was undertaken using the Gene Expression Profiling Interactive Analysis (ver. 2.0; GEPIA2) and miRTarBase databases to find out the overlapped genes which were upregulated in STAD and targeted by miR-328-3p (Figure 3(a)). As shown in Figure 3(b), the top 10 bottleneck proteins and top 10 hub proteins were selected by bottleneck and degree algorithm, respectively. Notably, through Cox regression analysis of the relationship between 34 overlapped genes and prognostic parameters, it was disclosed that CD44 was a prognostic factor for patients with STAD (Supplementary Table 3). Further, high expression of hub-bottleneck gene CD44 was associated with poor prognosis of STAD patients (Figure 3(c)). In addition, the results of GO enrichment analysis indicated 7 terms in the GO enrichment of the CD44 gene (Figure 3(d)), which mainly included positive regulation of cell proliferation, negative regulation of cell apoptosis, negative regulation of apoptotic signaling pathway, regulation of apoptotic signaling pathway, regulation of cell migration, and positive regulation of cell migration and pathways in cancer, indicating the involvement of CD44 in regulating STAD cell proliferation, apoptosis, and migration. By analyzing the expression level of CD44 in the GC dataset, we uncovered that CD44 was abundantly expressed in STAD (Figure S1A). Likewise, upregulation of CD44 in STAD cells was confirmed by qRT-PCR (Figure S1B). Therefore, CD44 was chosen for subsequent investigations.

In order to explore the correlation between miR-328-3p and CD44, we predicted their binding sites in the TarBase database. Dual-luciferase reporter assay revealed that miR-328-3p mimics remarkably lessened the luciferase activity of cells transfected with CD44-WT plasmids (*P* < 0.01), while there was no change in the luciferase activity of cells transfected with CD44-MUT plasmids (Figure 3(d)). Consistently, RIP experiment illuminated that forced expression of miR-328-3p promoted the enrichment of CD44 in AGO2 precipitates, providing solid evidence that miR-328-3p directly bound to CD44 (Figure 3(f)). Additionally, it was found that overexpression of miR-328-3p remarkably suppressed CD44 expression, while inhibiting miR-328-3p led to opposite effects (Figure 3(g)). Collectively, miR-328-3p directly inhibited CD44 in STAD cells, and CD44 was correlated with various cell activities in STAD.

### 3.4. miR-328-3p Worked as a Cancer Suppressor in STAD via Inhibiting CD44 Expression

**Figure 4:**
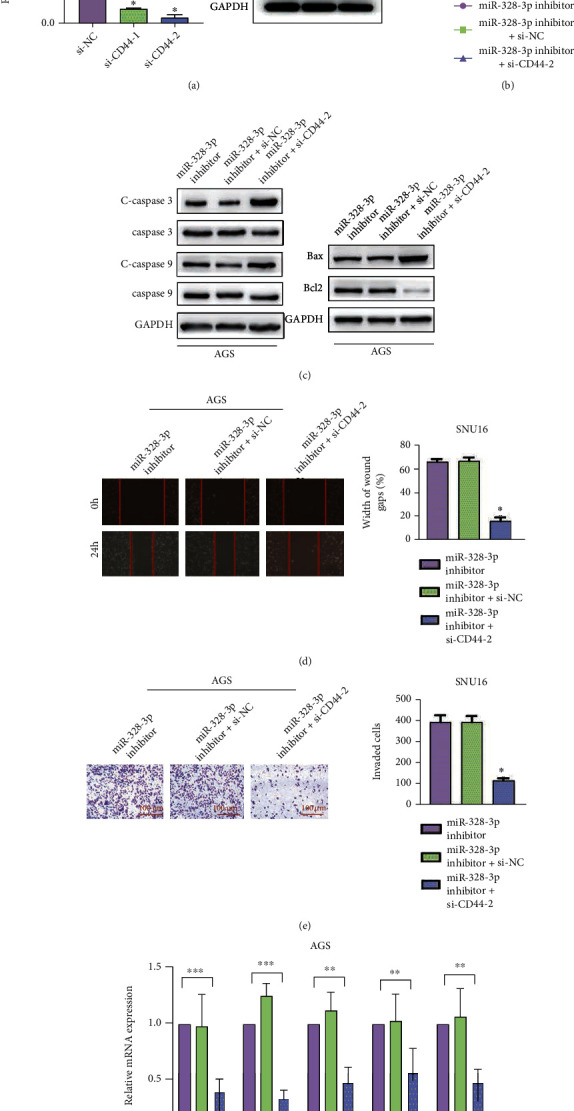
miR-328-3p worked as a cancer suppressor in STAD via targeting CD44. To further indicate whether miR-328-3p regulated the behaviors of STAD cells in a CD44-dependent manner, AGS cells were cotransfected with miR-328-3p inhibitors and si-CD44. (a) qRT-PCR and Western blot were utilized to detect the transfection efficiency of CD44 knockdown. (b) CCK-8 assay was used to assess the proliferation rate of AGS cells. (c) Western blot assay was used to detect the expression levels of apoptosis-related proteins. (d, e) The migration and invasion of AGS cells were assessed with (d) wound healing and (e) Transwell assays. (f) The expression levels of SOX2, NANOG, EPCAM, CD133, and CD166 were determined by qRT-PCR. Scale bar = 100 *μ*m. All experiments were performed in triplicate. ^∗^*P* < 0.05, ^∗∗^*P* < 0.01, and ^∗∗∗^*P* < 0.001.

Accordingly, we intended to verify whether miR-328-3p could regulate biological processes of STAD in a CD44-dependent manner. After siRNAs against CD44 (si-CD44#1 and si-CD44#2) or negative control si-NC was transfected into STAD cells, qRT-PCR and Western blot testified that AGS cells transfected with si-CD44#2 showed the lowest expression level of CD44 (Figure 4(a)). As a result, si-CD44#2 was used for further research. The results of CCK-8 assay demonstrated that inhibiting miR-328-3p remarkably enhanced the proliferation of AGS cells, which could be blocked by silencing CD44 (Figure 4(b)). In addition, decreased expression levels of Bax, cleaved caspase-3, and cleaved caspase-9 as well as enhanced expression of Bcl-2 were caused by downregulation of miR-328-3p, which could be rescued by knockdown of CD44 (Figure 4(c)). Furthermore, the effects of miR-328-3p inhibitors on AGS cell migration and invasion could be blocked by silencing CD44 (Figures 4(d) and 4(e)). In concert with these results, qRT-PCR revealed that knockdown of CD44 abated the promoting role of miR-328-3p inhibitors in the expression levels of SOX2, NANOG, EPCAM, CD133, and CD166 (Figure 4(f)). Collectively, miR-328-3p attenuated the malignancy of STAD via inhibiting CD44.

### 3.5. MZF1 Transcriptionally Activated miR-328-3p Expression in STAD Cells

**Figure 5:**
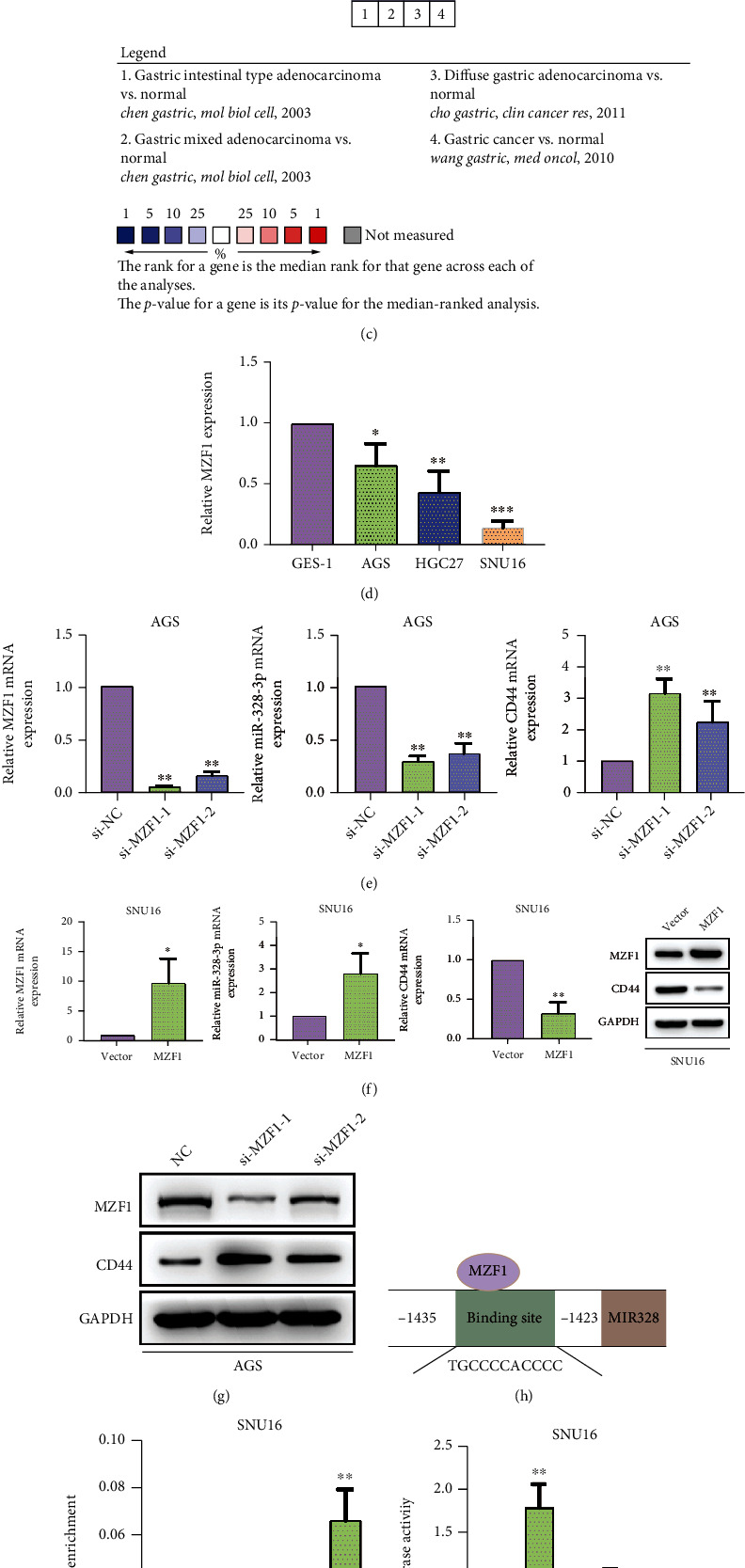
MZF1 activated the transcription of miR-328-3p. (a) PuTmiR and TransmiR databases were used to predict the transcription factor of miR-328-3p. MZF1, RREB1, BACH1, and STAT1 were four common transcription factors. (b) The expression levels of indicated four transcription factors in STAD tissues were analyzed. (c) The analysis of GC dataset in Oncomine database revealed MZF1 expression levels in STAD tissues. (d) qRT-PCR detection of MZF1 expression in STAD cells. (e, f) qRT-PCR assay was utilized to detect the expression levels of MZF1, miR-328-3p, and CD44 in (e) transfected AGS and (f) SNU16 cells. (g) Western blot assay was employed to detect the expression levels of MZF1 and CD44. (h) The TransmiR database was used to predict the binding sites of MZF1 in pre-miR-328-3p promoter regions. (i, j) The interaction between MZF1 and miR-328-3p was confirmed by (i) ChIP and (j) dual-luciferase reporter assays. All experiments were performed in triplicate. ^∗^*P* < 0.05, ^∗∗^*P* < 0.01, and ^∗∗∗^*P* < 0.001.

We predicted the transcription factor of miR-328-3p using PuTmiR and TransmiR databases, and a protein-protein interaction network was accordingly established (Figure 5(a)). It was found that there were four common genes in the network, including MZF1, RREB1, BACH1, and STAT1, among which only MZF1 was downregulated in STAD tissues (Figure 5(b)). Similarly, the analysis of the GC dataset suggested that MZF1 was weakly expressed in STAD tissues compared with normal samples (Figure 5(c)). Moreover, the low expression level of MZF1 was also validated in STAD cells (Figure 5(d)).

To further examine the interaction between MZF1 and miR-328-3p, we silenced MZF1 in AGS cells and found that knockdown of MZF1 efficiently suppressed miR-328-3p expression and increased the mRNA level of CD44 (Figure 5(e)). On the contrary, MZF1 was overexpressed in SNU16 cells, and then, our findings manifested that enhanced expression of MZF1 resulted in a significant increase in miR-328-3p expression and an overt reduction in CD44 mRNA expression (Figure 5(f)). In agreement with qRT-PCR, we demonstrated that the protein expression level of CD44 was elevated by silencing MZF1 and declined by upregulation of MZF1 (Figure 5(g)). In addition, it was noted that MZF1 motif could bind to the promoter regions of miR-328-3p (Figure 5(h)). ChIP assay revealed that anti-MZF1 antibody enriched more DNA fragments of miR-328-3p promoter region compared with that in the IgG control group. More importantly, overexpression of MZF1 contributed to the enrichment of miR-328-3p promoter by anti-MZF1 antibody (Figure 5(i)). The results of dual-luciferase reporter assay exhibited that upregulation of MZF1 efficiently enhanced the luciferase activity of cells transfected with miR-328-3p-WT plasmids (*P* < 0.01), while there was no change in the luciferase activity of cells transfected with miR-328-3p-MUT (Figure 5(j)). These data suggested that MZF1 induced the activation of miR-328-3p transcription through binding to its promoter region.

### 3.6. MZF1 Inhibited the Malignancy of STAD through Activating miR-328-3p Expression

**Figure 6:**
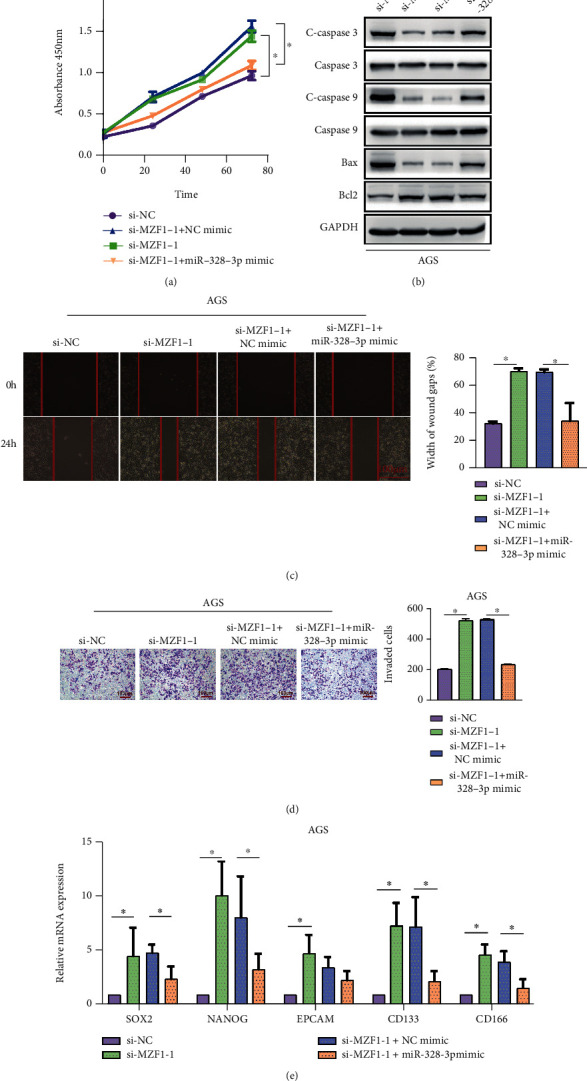
MZF1 inhibited the progression of STAD via promoting miR-328-3p expression. To further indicate whether MZF1 could affect STAD development in a miR-328-3p-dependent manner, AGS cells were cotransfected with si-MZF1 and miR-328-3p mimics. (a) CCK-8 assay was used to evaluate the proliferation rate of AGS cells. (b) Western blot assay was performed to detect the expression levels of apoptosis-related proteins. (c, d) The migration and invasion of AGS cells were assessed with (c) wound healing and (d) Transwell assays. (e) qRT-PCR analysis of the expression levels of stem landmarks. Scale bar = 100 *μ*m. All experiments were performed in triplicate. ^∗^*P* < 0.05, ^∗∗^*P* < 0.01, and ^∗∗∗^*P* < 0.001.

To further confirm whether MZF1 could affect STAD cell behaviors in a miR-328-3p-dependent manner, cotransfection of AGS cells with si-MZF1 and miR-328-3p-mimics was performed. Besides, the results of CCK-8 assay (Figure 6(a)) showed that silencing of MZF1 remarkably accelerated the proliferation of AGS cells, which could be blocked by miR-328-3p overexpression. In addition, silencing of MZF1 increased Bcl-2 protein levels and inhibited the expression of Bax, cleaved caspase-3, and cleaved caspase-9, which could be efficiently rescued by upregulation of miR-328-3p (Figure 6(b)). Moreover, enhanced migratory and invasive abilities of AGS cells caused by knockdown of MZF1 were recovered by miR-328-3p overexpression (Figures 6(c) and 6(d)). Consistently, we observed that silencing MZF1 weakened the mRNA expression of SOX2, NANOG, EPCAM, CD133, and CD166, while miR-328-3p mimics abolished the regulatory impacts of si-MZF1 on stem landmarks (Figure 6(e)). These findings indicated that MZF1 suppressed the development of STAD through activating the miR-328-3p expression.

### 3.7. miR-328-3p Inhibited the Growth of STAD Cells *In Vivo*

**Figure 7:**
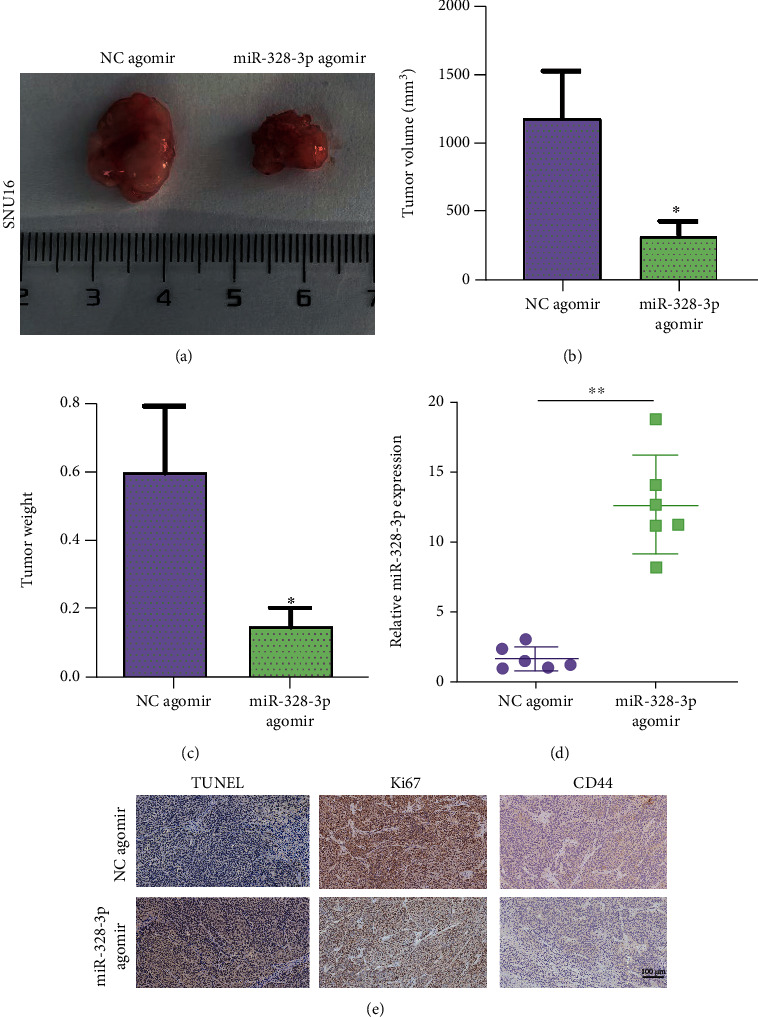
miR-328-3p inhibited tumor growth of STAD *in vivo*. SNU16 cells transfected with NC agomir or miR-328-3p agomir were subcutaneously injected into nude mice for observation of tumor growth. (a) The images of tumor tissues from the NC agomir and miR-328-3p agomir groups. (b) Tumor volume. (c) Tumor weight. (d) qRT-PCR assay was utilized to detect the expression level of miR-328-3p in xenografts. (e) IHC was employed to measure the expression levels of CD44 and Ki67 in xenograft tumors, and TUNEL staining was used to measure apoptotic cells in tumors. All experiments were performed in triplicate. ^∗^*P* < 0.05, ^∗∗^*P* < 0.01.

To further validate the functional effects of miR-328-3p *in vivo*, the control and miR-328-3p-upregulated SNU16 cells were subcutaneously injected into nude mice. The results showed that tumor growth of nude mice injected with miR-328-3p-upregulated SNU16 cells was slower than that of the control mice (Figures 7(a) and 7(b)). Additionally, overexpression of miR-328-3p caused the conspicuous reduction of tumor weight (Figure 7(c)), and the miR-328-3p expression level was significantly upregulated in tumors from the miR-328-3p-agomir group compared with the control group (Figure 7(d)). Moreover, the results of IHC staining revealed that the expression levels of CD44 and Ki-67 were remarkably downregulated in xenografts formed by mice in the miR-328-3p-agomir group. Consistent with the above findings, TUNEL staining manifested that the number of apoptotic cells in tumor tissues from the miR-328-3p-agomir group was larger compared to the control group (Figure 7(f)). Taken together, miR-328-3p remarkably suppressed *in vivo* tumor growth of STAD.

## 4. Discussion

In the present study, our results indicated that miR-328-3p was downregulated in STAD tissues and cells, and miR-328-3p overexpression suppressed the malignant features of STAD cells, while inhibiting miR-328-3p produced the opposite results. Moreover, CD44 was found as the target gene of miR-328-3p, and MZF1 was considered an upstream transcription factor for miR-328-3p. In addition, it was validated that MZF1 suppressed the malignancy of STAD via activating the miR-328-3p/CD44 axis.

A previous study reported that the miR-328-3p expression level was downregulated in STAD tissues [12], which was in concert with our results, indicating that miR-328-3p was highly associated with STAD progression. In addition, several studies show that miR-328-3p plays a crucial role in the mediation of cellular activities [30], tumor metastasis [31], and inflammation [32]. Consistent with previously reported findings, the current study revealed that miR-328-3p inhibited cell proliferation, migration, invasion, and stemness and promoted cell apoptosis in STAD. More importantly, tumor xenograft assay further confirmed the suppressive function of miR-328-3p in the growth of STAD cells *in vivo*. Namely, our results indicated that miR-328-3p acted as a tumor suppressor in the progression of STAD, confirming the regulatory role of miR-328-3p in STAD.

As a glycoprotein, a high expression level of CD44 is associated with poor prognosis of cancer patients, including head and neck squamous cell carcinoma (HNSCC) [33], glioblastoma [34], and hepatocellular carcinoma [35]. Through bottleneck and degree algorithm, CD44 was identified as a hub-bottleneck gene. Besides, Cox regression analysis demonstrated that CD44 was a prognostic factor for STAD patients. Consistent with previous studies, it was found that a high expression level of CD44 predicted a poor overall survival of STAD patients. In addition, GO enrichment analysis suggested that CD44 mediated the proliferation, apoptosis, and migration of STAD cells. Notably, we further validated the targeting relationship between miR-328-3p and CD44 and explored the role of CD44 in miR-328-3p-mediated STAD through cellular experiments. It was certified that miR-328-3p impeded the progression of STAD via inhibiting CD44.

Although CD44 is identified as the target gene of miR-328-3p, the upstream regulator of miR-328-3p remains to be studied. Thus, with incorporation of the results of bioinformatics analysis and cellular experiments, we considered MZF1 as a transcription activator of miR-328-3p. To date, a number of studies have presented a complicated interaction network for the regulated mechanism of MZF1 and its biofunctional role in multiple types of cancers. For instance, Lee et al. reported that Ik-1 and MZF1 acted as inhibitors to suppress the viability, metastasis, and anchorage-independent colony formation of lymphoma via downregulating IGF-IR expression [36]. In contrast, Tsai et al. suggested that LKB1 loss-mediated MZF1 induced the transcription of MYC, thereby driving the progression of lung adenocarcinoma [37]. Our findings clearly indicated that MZF1 directly bound to miR-328-3p promoter and activated its expression in STAD cells. Moreover, it was found that the promoting effects of MZF1 knockdown on the malignant behaviors of STAD cells by were blocked by overexpression of miR-328-3p.

In summary, our study revealed that miR-328-3p was downregulated in STAD and retarded the development of STAD via inhibiting CD44. Moreover, it was found that miR-328-3p expression level was upregulated by MZF1 in a transcriptional activation-dependent manner, and MZF1 suppressed the progression of STAD. These findings suggested the MZF1/miR-328-3p/CD44 axis may be considered a novel therapeutic strategy against progression of STAD.

## Data Availability

All data generated or analyzed during this study are included in this published article.

## Supplementary Materials

Supplementary Materials Supplementary Table 1: sequences of siRNA against specific targets. Supplementary Table 2: sequences of PCR primers used in this study. Supplementary Table 3: Cox regression analysis of the relationship between 34 overlapped genes and prognostic parameters. Supplementary Figure 1: CD44 expression levels in STAD tissues and cells. (A) The analysis of GC dataset in Oncomine database revealed the expression level of CD44 in STAD tissues. (B) qRT-PCR and Western blot detection of CD44 expression level in STAD and normal cells. All experiments were performed in triplicate. ^∗^*P* < 0.05, ^∗∗^*P* < 0.01.
