# Supplementary material for: MZF1 Transcriptionally Activated MicroRNA-328-3p Suppresses the Malignancy of Stomach Adenocarcinoma via Inhibiting CD44
Source: J Immunol Res. 2022 May 28;2022:5819295. doi: 10.1155/2022/5819295 (PMC9167131; doi:10.1155/2022/5819295)
Supplement: Supplementary Materials — Supplementary Table 1: sequences of siRNA against specific targets. Supplementary Table 2: sequences of PCR primers used in this study. Supplementary Table 3: Cox regression analysis of the relationship between 34 overlapped genes and prognostic parameters. Supplementary Figure 1: CD44 expression levels in STAD tissues and cells. (A) The analysis of GC dataset in Oncomine database revealed the expression level of CD44 in STAD tissues. (B) qRT-PCR and Western blot detection of CD44 expression level in STAD and normal cells. All experiments were performed in triplicate. ∗P < 0.05, ∗∗P < 0.01. [file 5819295.f1.zip › 5819295.f1/Supplementary Figure 1.pdf]

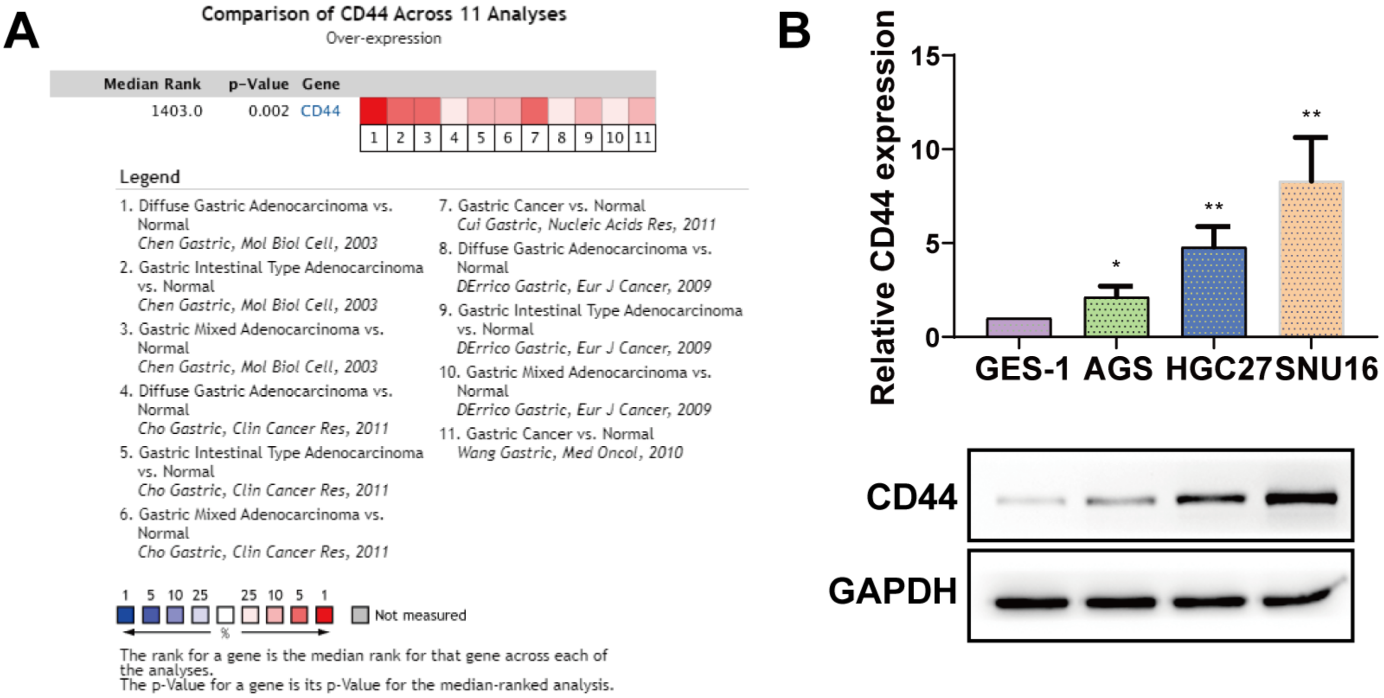
A
Comparison of CD44 Across 11 Analyses
Over-expression
Median Rank p-Value Gene
1403.0 0.002 CD44
1 2 3 4 5 6 7 8 9 10 11
Legend
1. Diffuse Gastric Adenocarcinoma vs. Normal
Chen Gastric, Mol Biol Cell, 2003
2. Gastric Intestinal Type Adenocarcinoma vs. Normal
Chen Gastric, Mol Biol Cell, 2003
3. Gastric Mixed Adenocarcinoma vs. Normal
Chen Gastric, Mol Biol Cell, 2003
4. Diffuse Gastric Adenocarcinoma vs. Normal
Cho Gastric, Clin Cancer Res, 2011
5. Gastric Intestinal Type Adenocarcinoma vs. Normal
Cho Gastric, Clin Cancer Res, 2011
6. Gastric Mixed Adenocarcinoma vs. Normal
Cho Gastric, Clin Cancer Res, 2011
7. Gastric Cancer vs. Normal
Cui Gastric, Nucleic Acids Res, 2011
8. Diffuse Gastric Adenocarcinoma vs. Normal
DErrico Gastric, Eur J Cancer, 2009
9. Gastric Intestinal Type Adenocarcinoma vs. Normal
DErrico Gastric, Eur J Cancer, 2009
10. Gastric Mixed Adenocarcinoma vs. Normal
DErrico Gastric, Eur J Cancer, 2009
11. Gastric Cancer vs. Normal
Wang Gastric, Med Oncol, 2010
1 5 10 25 25 10 5 1
%
Not measured
The rank for a gene is the median rank for that gene across each of the analyses.
The p-Value for a gene is its p-Value for the median-ranked analysis.
B
Relative CD44 expression
15
10
5
0
*
**
**
GES-1
AGS
HGC27
SNU16
CD44
GAPDH
