# Supplementary material for: MZF1 Transcriptionally Activated MicroRNA-328-3p Suppresses the Malignancy of Stomach Adenocarcinoma via Inhibiting CD44
Source: J Immunol Res. 2022 May 28;2022:5819295. doi: 10.1155/2022/5819295 (PMC9167131; doi:10.1155/2022/5819295)
Supplement: Supplementary Materials — Supplementary Table 1: sequences of siRNA against specific targets. Supplementary Table 2: sequences of PCR primers used in this study. Supplementary Table 3: Cox regression analysis of the relationship between 34 overlapped genes and prognostic parameters. Supplementary Figure 1: CD44 expression levels in STAD tissues and cells. (A) The analysis of GC dataset in Oncomine database revealed the expression level of CD44 in STAD tissues. (B) qRT-PCR and Western blot detection of CD44 expression level in STAD and normal cells. All experiments were performed in triplicate. ∗P < 0.05, ∗∗P < 0.01. [file 5819295.f1.zip › 5819295.f1/Supplementary tables.pdf]

**Supplementary Table 1. Sequences of siRNA Against Specific Targets**

| | | |
|---|---|---|
| si-CD44-1 | 5'-3' | CCCAGTATGACACATATTGCTTC |
| si-CD44-2 | 5'-3' | ATCTTTTACACCTTTTCTACTGT |
| si-MZF1-1 | 5'-3' | CCCAGTATGACACATATTGCTTC |
| si-MZF1-2 | 5'-3' | TGGTTTTCATGGTTGTTTCTACC |

**Supplementary Table 2. Sequences of PCR primers used in this study**

| | | |
|---|---|---|
| CD44 | Forward(5'-3') | CTGCCGCTTTGCAGGTGTA |
| | Reverse(5'-3') | CATTGTGGGCAAGGTGCTATT |
| hsa-miR-328-3p | Forward(5'-3') | GCCCTCTCTGCCCTTC |
| | Reverse(5'-3') | GTCCAGTTTTTTTTTTTTTTTACGGA |
| U6 | Forward(5'-3') | CGCTTC ACGAATTTGCGTGTCAT |
| | Reverse(5'-3') | GCTTCGGCAGCACATATACTAAAAT |
| GAPDH | Forward(5'-3') | GGAGCGAGATCCCTCCAAAAT |
| | Reverse(5'-3') | GGCTGTTGTCATACTTCTCATGG |

**Supplementary Table 3. Cox regression analysis of the relationship between 34 overlapped genes and prognostic parameters**

| Characteristics | Total(N) | HR(95% CI) Univariate analysis | P value Univariate analysis | HR(95% CI) Multivariate analysis | P value Multivariate analysis |
|---|---|---|---|---|---|
| ADNP | 349 | 1.080 (0.795-1.466) | 0.623 | | |
| AEBP2 | 349 | 1.270 (0.919-1.755) | 0.148 | | |
| AGO1 | 349 | 1.049 (0.773-1.423) | 0.759 | | |
| ALDH1B1 | 349 | 0.993 (0.820-1.203) | 0.942 | | |
| APP | 349 | 0.985 (0.727-1.336) | 0.925 | | |
| ARL6IP1 | 349 | 1.104 (0.839-1.453) | 0.478 | | |
| CBX4 | 349 | 1.181 (0.911-1.530) | 0.21 | | |
| CD44 | 349 | 1.197 (1.007-1.423) | 0.041 | 1.204(1.012-1.432) | 0.036 |
| DYNLL1 | 349 | 1.615 (0.977-2.670) | 0.062 | | |
| GDI2 | 349 | 0.847 (0.573-1.251) | 0.404 | | |
| PKM | 349 | 1.053 (0.814-1.363) | 0.693 | | |
| POLR3G | 349 | 0.870 (0.665-1.140) | 0.313 | | |
| RAD51 | 349 | 0.887 (0.687-1.146) | 0.36 | | |
| SCD | 349 | 1.064 (0.929-1.219) | 0.369 | | |
| SLC25A40 | 349 | 0.953 (0.668-1.358) | 0.789 | | |

| | | | |
|---|---|---|---|
| SMC1A | 349 | 0.801 (0.583-1.100) | 0.171 |
| TMEM33 | 349 | 0.874 (0.637-1.199) | 0.404 |
| TOMM40 | 349 | 0.955 (0.737-1.239) | 0.731 |
| H2AX | 349 | 0.936 (0.744-1.177) | 0.572 |
| HMGB1 | 349 | 0.858 (0.585-1.259) | 0.435 |
| HNRNPF | 349 | 0.753 (0.531-1.067) | 0.11 |
| IRAK1 | 349 | 0.809 (0.625-1.048) | 0.109 |
| MKI67 | 349 | 0.932 (0.771-1.126) | 0.466 |
| RAB43 | 349 | 0.982 (0.716-1.348) | 0.912 |
| SCAMP2 | 349 | 0.968 (0.687-1.365) | 0.855 |
| SERBP1 | 349 | 0.855 (0.569-1.285) | 0.452 |
| SKA1 | 349 | 0.887 (0.715-1.102) | 0.279 |
| SLC7A1 | 349 | 0.869 (0.672-1.124) | 0.285 |
| STT3A | 349 | 0.940 (0.675-1.309) | 0.716 |
| TAPBP | 349 | 1.057 (0.773-1.446) | 0.729 |
| TMOD3 | 349 | 1.150 (0.799-1.655) | 0.45 |
| VPS53 | 349 | 0.986 (0.668-1.457) | 0.945 |
| YWHAQ | 349 | 1.096 (0.743-1.616) | 0.644 |
| ZC3H12D | 349 | 0.936 (0.738-1.185) | 0.582 |
